# A Meta-Analysis of Randomized Clinical Trials of Runzao Zhiyang Capsule in Chronic Urticaria

**DOI:** 10.1155/2022/1904598

**Published:** 2022-09-17

**Authors:** Shengzhen Ye, Xueer Zhang, Guihua Ling, Xianjun Xiao, Dan Huang, Mingling Chen

**Affiliations:** ^1^Chengdu University of Traditional Chinese Medicine, Chengdu 610075, Sichuan Province, China; ^2^Affiliated Hospital of Chengdu University of Traditional Chinese Medicine, Chengdu 610072, Sichuan Province, China

## Abstract

Chinese herbal medicine has many advantages in the treatment of chronic urticaria (CU). Herein, we evaluated the efficacy and safety of the Runzao Zhiyang (RZZY, Chinese patent herbal medicine capsule) capsule for CU through a meta-analysis of randomized clinical trials (RCTs). This meta-analysis included 17 RCTs involving 1,760 patients. RZZY capsule combined with conventional drugs showed a better clinical total effective rate (risk ratio (RR) = 1.20, 95% confidence interval (CI) (1.15, 1.24), *P* < 0.00001), significantly reduced the adverse reaction rate [RR = 0.68, 95% CI (0.50, 0.92), *P*=0.01] and recurrence rate [RR = 0.29, 95% CI (0.18, 0.46), *P* < 0.00001], and improved the life quality of patients (mean difference (MD) = −2.95, 95% CI (−4.32, −1.57), *P*=0.0001). Meanwhile, the serum Interleukin-4 (IL-4) (MD = −13.83, 95% CI (−23.45, −4.20), *P*=0.005) and immunoglobulin *E* (IgE) (MD = −22.99, 95% CI (−31.48, −14.50), *P* < 0.00001) of patients in the intervention group decreased more significantly. In all, the RZZY capsule has potential therapeutic advantages and is relatively safe for CU. However, we are cautious about the conclusion, which needs to be further confirmed by more large samples, multicenter, and high-quality research in the later stage.

## 1. Introduction

Chronic urticaria (CU) is a common skin disorder characterized by recurring wheals or combined with angioedema, accompanied by an itching or burning sensation, which occurs repeatedly and lasts for more than 6 weeks. The skin usually returns to its normal appearance within one day after each attack [1, 2]. According to statistics, CU affects 1% of the world's population, mainly youthful and middle-aged females, and it is more common in Asia than in Europe and North America [3, 4]. Many patients with CU have anxiety, depression, sleep disorders, autoimmune thyroid diseases, and other comorbidities [5–7]. CU patients with low quality of life make hospitals and society bear great economic pressure [3, 8, 9].

The etiology of CU is unknown and rarely caused by allergen-mediated type I allergy. Studies have shown that histamine is the main mediator of CU. Therefore, the guideline recommends that 2^nd^-generation H_1_ antihistamines (such as Ebastine, Cetirizine, and Loratadine) are the first-line therapy, while the second-line treatment is by increasing their dose [10]. However, relevant studies have reported that even at higher doses, more than a quarter of cases are resistant to H_1_ antihistamines. For CU patients who did not respond to the 2^nd^-generation H_1_ antihistamines, the treatment of biological agents (such as Omalizumab) was proposed [10, 11]. Nevertheless, the price of biological agents is relatively expensive, and the long-term risk is not clear, so it is difficult to use widely. In addition, the immunosuppressants (such as Cyclosporine A) can also be applied to patients who are insensitive or ineffective to the 2^nd^-generation H_1_ antihistamines. However, due to the high incidence of adverse reactions to immunosuppressants, it is not recommended as a standard treatment [12, 13].

Chinese herbal medicine (CHM) is a great treasure of China. After millennium development, its therapeutic advantages have been gradually explored. Many Chinese herbal medicines (such as Runzao Zhiyang (RZZY) capsule, Danggui Yinzi, Siwu decoction, and Xiaofeng powder) have been proven to be effective in relieving symptoms of CU [14–18]. In the theoretical system of Chinese medicine, CU is related to wind evil outside and deficiency of yin (The Yinyang concept is the basic concept of Chinese philosophy as well as traditional Chinese medicine) and blood inside Therefore, the treatment of moistening dryness and enriching the blood, dispelling wind, and arresting itching should be adopted. The RZZY capsule is approved by the China National Medical Products Administration and has the functions of nourishing yin and blood, dispelling wind, relieving itching, moistening intestines, and defecating. At present, several randomized controlled trials (RCTs) [19–35] show that the RZZY capsule has better clinical efficacy in treating CU.

The RZZY capsule consists of six kinds of traditional Chinese medicine (including *Polygonum multiflorum*, *Radix polygoni multiflori preparata*, *Rehmannia glutinosa*, *mulberry leaves*, *Sophora flavescens,* and *red live hemp*). From the perspective of modern pharmacology, *Polygonum multiflorum* has the effects of improving immunity, anti-inflammatory, antibacterial, and adrenocortical hormones [36, 37]. *Radix polygoni multiflori preparata* is a processed product of *Polygonum multiflorum*. Through animal experiments, researchers have proved that the polysaccharide, the main pharmacodynamic component of *Radix polygoni multiflori preparata*, has an immunomodulatory effect. It can ameliorate the learning and memory situation of dementia mice and reduce the content of total cholesterol and low-density lipoprotein in rat blood [38]. *Rehmannia glutinosa* can inhibit the release of cytokines and inflammatory mediators with antioxidant and anticancer effects [39–42]. *Mulberry leaves* can have anti-inflammation, immune regulation, liver protection, antianxiety, antidopamine, and hypoglycemic effects [43, 44]. Matrine, a component of *Sophora flavescens*, can inhibit the degranulation of mast cells and reduce the release of allergic mediators [21]. Su et al. conducted an in vitro experiment and proved that *red live hemp* has anti-inflammatory and analgesic effects, inhibits *T* lymphocyte proliferation, and inhibits the secretion of IL-2 and IFN-*γ* [45]. In addition, an animal experiment has demonstrated that the total coumarin, as the main chemical component of *red live hemp*, can regulate the expression of proinflammatory/anti-inflammatory cytokines in mice and can be used for developing immunosuppressive drugs [46].

The RZZY capsule has been widely used in the CU market of China and has achieved good curative effects. Therefore, several medical institutions have also conducted clinical research on it. However, the relatively small clinical samples and the lack of systematic and comprehensive evaluation exist in the single RCT, which is not enough to be effective evidence for the clinical application of the RZZY capsule. Thus, we conducted a meta-analysis, systematically analyzing and summarizing previous studies. Compared with the previous single study, by integrating all relevant studies, we can more accurately estimate the effect of the RZZY capsule on CU. This study is conducive to exploring the consistency of single-study evidence and the differences between studies and providing better evidence-based medical evidence for the RZZY capsule on CU.

## 2. Methods

### 2.1. Data Sources and Search Strategy

China National Knowledge Infrastructure (CNKI), Wanfang Data, VIP database, SinoMed, PubMed, EMbase, Cochrane Library, and Web of Science were searched for RCTs of RZZY capsule or combined with conventional drug for CU from the inception to July 30, 2021. There was no limitation on language. We searched using diﬀerent combinations of keywords, including “urticaria,” “chronic urticaria,” “Runzao Zhiyang capsule,” and “randomized clinical trials.” The search strategies of PubMed and CNKI are displayed in supplementary materials, respectively.

### 2.2. Study Registration and Inclusion/Exclusion Criteria

Study registration: this study followed the PRISMA statement [47], and the protocol was registered at PROSPERO (NO: CRD42021274429). The inclusion criteria were as follows: (1) type of study: the RCTs were qualified. (2) Participants: participants had been diagnosed with CU according to the Chinese guidelines for the diagnosis and treatment of urticaria (2018 Edition) [48]. (3) Intervention and comparison: the patients in the intervention group were treated with RZZY capsules combined with conventional drugs, while the patients in the control group were treated with conventional drugs (the course of treatment in both groups was at least 4 weeks). The exclusion criteria were as follows: (1) case report, animal experiment, experience summary, and other research types; (2) repeated publications; (3) unable to obtain full-text literature; (4) the research design is not rigorous; and (5) unable to extract valid outcome data or inconsistent outcome indicators.

### 2.3. Outcomes

1. Primary outcome: clinical total effective rate, which is defined as number of cured cases + markedly effective cases + effective cases/total cases × 100%; and adverse reaction rate.
2. Secondary outcomes: recurrence rate; Dermatology Life of Quality Index (DLQI); serum interleukin-4 (IL-4); and immunoglobulin E (IgE).

### 2.4. Study Selection and Data Extraction

Two researchers (X. E. Zhang and G. H. Ling) independently screened and cross-checked the articles. The researchers conducted preliminary screening through reading topics and abstracts to exclude the articles that cannot be included. By reading the full text and rescreening, the articles finally included in the meta-analysis were finally determined. Data extractions were performed by two independent reviewers (X. E. Zhang and G. H. Ling). It mainly included the first author, published year, sample size, age, gender, intervention, control measures, and outcome indicators. Any disagreement can be solved by consensus or by a third researcher (M. L. Chen).

### 2.5. Methodological Quality Assessment

The methodological quality of each study was evaluated by two authors (S. Z. Ye and D. Huang) using the Cochrane Handbook for Systematic Review of Intervention Version 5.1.0 [49]. It included the following items: random sequence generation, allocation concealment, blinding of participants and personnel, blinding of outcome assessment, incomplete outcome data, selective reporting, and other bias. Each item makes a judgment of low risk, high risk, or unclear risk according to the literature. Any disagreement can be solved by consensus or by a third researcher (M. L. Chen).

### 2.6. Statistical Analysis

RevMan 5.3 software was used for meta-analysis. Mean difference (MD) and risk ratio (RR) were evaluated for continuous data and dichotomous data, respectively. The statistical heterogeneity of the study was determined by the standard chi-square test and *I*^2^ statistics. When the risk of heterogeneity between studies is low, the chi-square test *P* ≥ 0.10 and *I*^2^ ≤ 50%, the fixed effect model should be used. Otherwise, the random effect model will be used.

### 2.7. Certainty of Evidence

Grading of Recommendations, Assessment, Development, and Evaluation (GRADE) assesses the certainty of evidence, considering five reasons: (1) risk of bias; (2) inconsistency; (3) indirectness; (4) imprecision; and (5) publication bias. The GRADE Pro Guideline Development Tool (GDT) online (https://gradepro.org) was used to create the summary of findings table.

## 3. Results

### 3.1. Description of Included Studies

**Figure 1:**
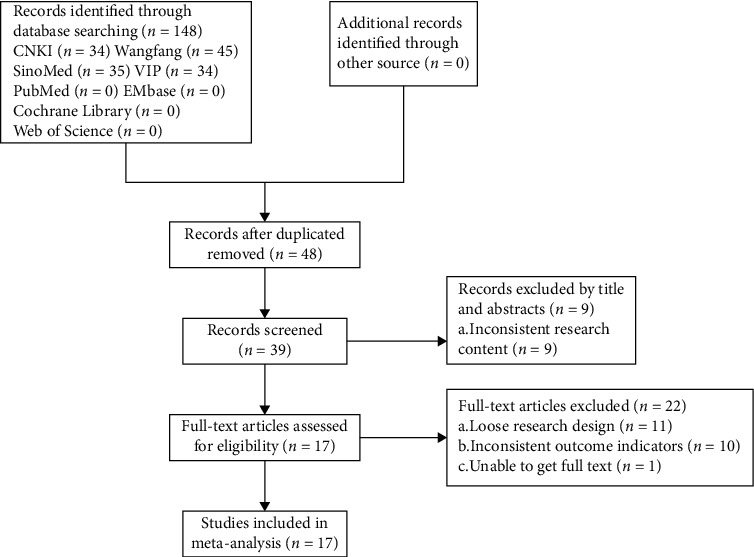
Flow diagram of study selection process.

148 related articles were retrieved. Among them, 100 duplicate articles were excluded, and 39 studies were obtained after reading the titles and abstracts. After screening according to the relevant criteria, 17 studies were finally included in the meta-analysis.  Figure 1 shows the flow chart of the study selection process.

### 3.2. Study Characteristics

All the RCTs were published in China from 2013 to 2020, involving a total of 1,760 participants. Before treatment, there was no significant difference in age, gender, and course of disease between the two groups. Patients in the control group were treated with simple conventional drugs, and the patients in the intervention group were treated with RZZY capsules based on the control group. The duration of treatment for both intervention and control groups ranged from 4 to 8 weeks. 17 trails [19–35] reported the total clinical effective rate, 14 studies [19, 21–24, 26–32, 34, 35] covered the adverse reactions rate, 6 RCTs [19, 27, 28, 30, 31, 35] studied the recurrence rate, 3 cases [19, 21, 33] appraised the changes of DLQI, and 5 studies [21, 22, 28, 31, 32] evaluated the changes of serological indexes before and after treatment. The characteristics of all included studies are summarized in Table 1.

### 3.3. Risk of Bias Assessment

**Figure 2:**
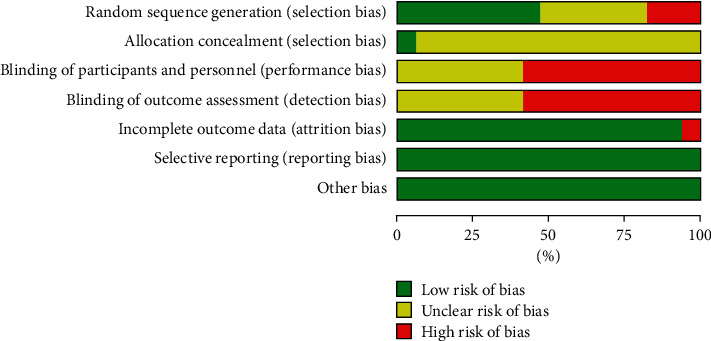
Risk of bias graph.

**Figure 3:**
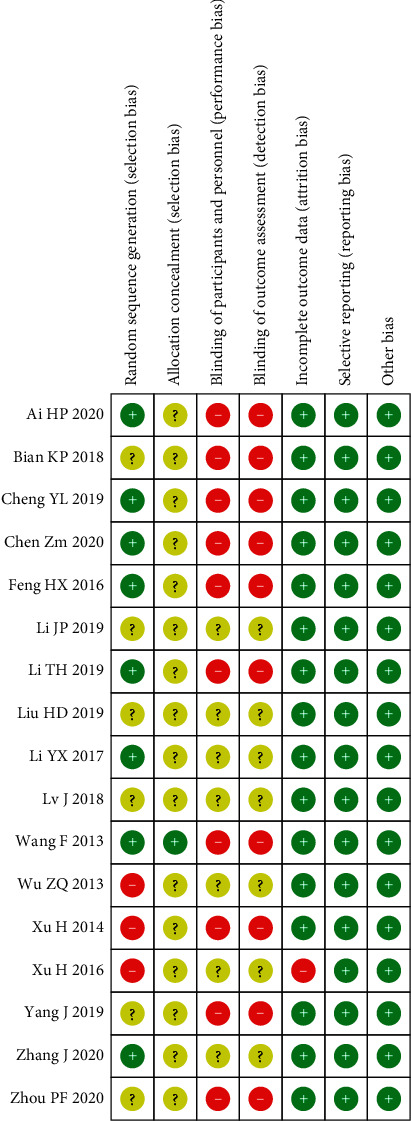
Risk of bias summary.

All 17 RCTs [19–35] mentioned random methods, of which 11 [20–24, 30–35] mentioned specific random methods (8 [20–24, 30, 31, 35] of them are low risk) and 6 [19, 25–29] only mentioned the word “random” without any description in detail. One study [35] used SAS system random number table allocation, and other studies did not mention allocation concealment. 7 studies [20, 25, 27, 29, 30, 32, 34] did not explain whether the blind method was used, and the rest did not use the blind method. 2 cases were lost to follow-up in one study [32], and there was no case shedding in other studies. Both primary and secondary outcome indicators were reported, and there was no risk of selective reporting or other bias. The risk of bias is summarized in Figures 2 and 3 and Table 2.

### 3.4. Meta-Analysis

#### 3.4.1. Total Effective Rate

**Figure 4:**
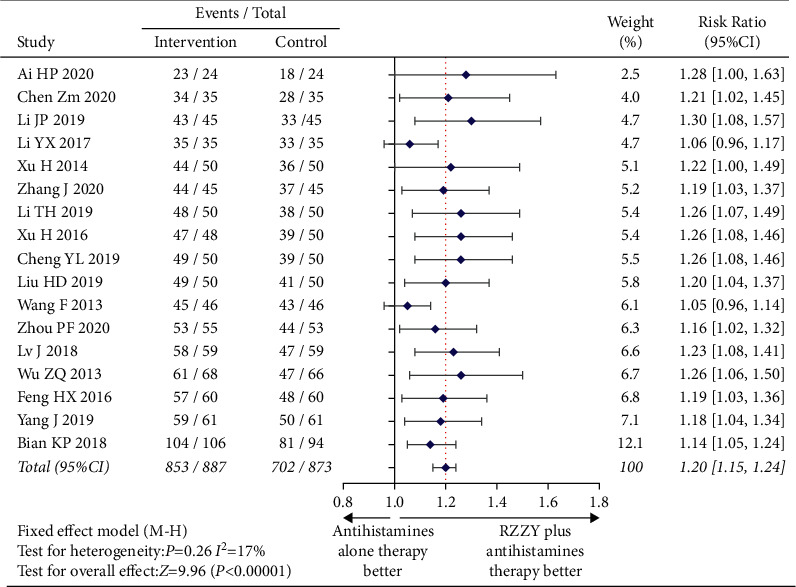
Risk ratio of total effective rate.

All the 17 trials [19–35] reported total effective rates. The results showed that the total effective rate of the RZZY capsule combined with antihistamines was significantly better than that of antihistamines alone (RR = 1.20, 95% CI [1.15,1.24, *I*^2^ = 17%, *P* < 0.00001), as shown in Figure 4. This indicated a 1.20 times improvement of combination patients over that of antihistamines alone patients for clinical efficacy, which can be statistically explained by the fact that the clinical efficacy of the combined drug group is better than that of the simple drug group.

#### 3.4.2. Adverse Reaction Rate

**Figure 5:**
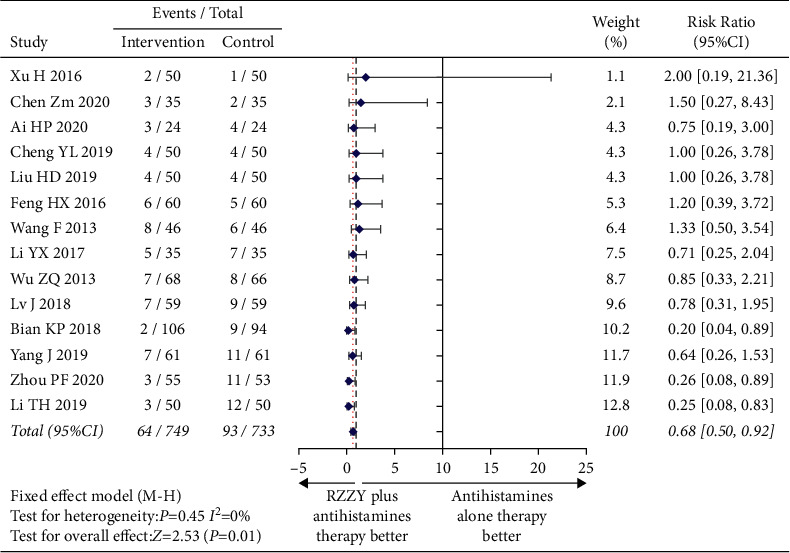
Risk ratio of adverse reaction rate.

No serious adverse events were reported in 14 studies [19, 21–24, 26–32, 34, 35]. The adverse reactions of the two groups mainly included drowsiness, dizziness, fatigue, dry mouth, stomach discomfort, loose stools, and constipation, which disappeared after drug withdrawal. Four studies [27, 29, 30, 35] conducted laboratory tests on two groups of patients and the results showed that there were no abnormalities in blood routine, urine routine, liver and kidney function, or electrocardiogram in both groups. Upon meta-analysis, as shown in Figure 5, the results showed that the adverse reaction rate in the intervention group was significantly lower than that in the control group (RR = 0.68, 95% CI (0.50,0.92), *I*^2^ = 0%, *P*=0.01).

#### 3.4.3. Recurrence Rate

**Figure 6:**
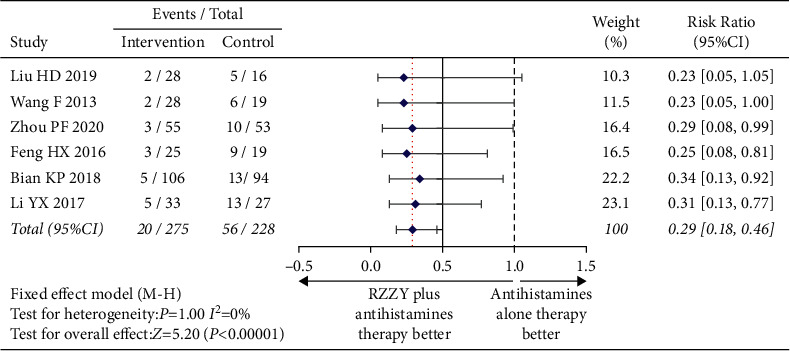
Risk ratio of recurrence rate.

As suggested by six articles [19, 27, 28, 30, 31, 35] involving 690 patients, the recurrence rate of the group combined with RZZY capsules was significantly lower than that of the group using antihistamines alone (RR = 0.29, 95% CI [0.18, 0.46], *I*^2^ = 0%, *P* < 0.00001) (Figure 6).

#### 3.4.4. Quality of Life

**Figure 7:**
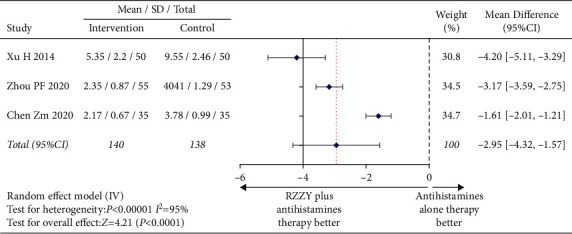
Mean difference of DLQI.

The quality of life was evaluated by DLQI. The pooled data of 3 RCTs [19, 21, 33] reported the improvement of quality of life in patients with CU after treatment. It is obtained by the random effect model that the improvement of quality of life in the intervention group was better than that in the control group (MD = −2.95, 95% CI −4.32, −1.57], *I*^2^ = 95%, *P* < 0.00001) (Figure 7).

#### 3.4.5. IL-4 and IgE Level

**Figure 8:**
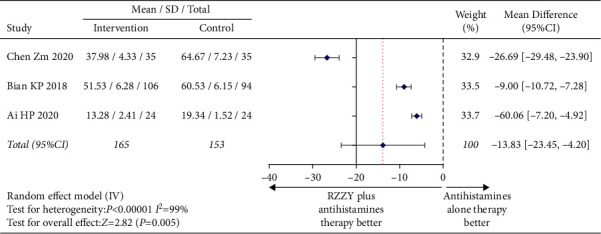
Mean difference of IL-4.

**Figure 9:**
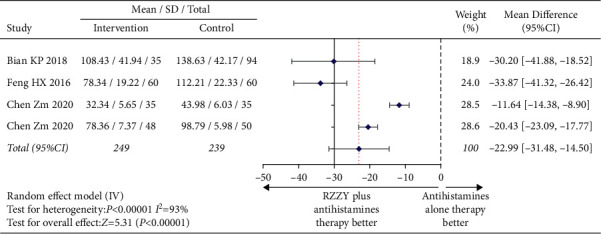
Mean difference of IgE.

The changes of serum IL-4 level before and after treatment were evaluated by three studies [21, 22, 28]. The pooled data demonstrated that the level of serum IL-4 in the RZZY capsule group decreased more significantly than that in the antihistamines group after treatment (MD = −13.83, 95% CI [−23.45, −4.20], *I*^2^ = 99%, *P*=0.005) (Figure 8). Changes in IgE levels were mentioned in four studies [21, 28, 31, 32]. The analysis showed that IgE in the intervention group decreased faster after 4 or 8 weeks of treatment (MD = −22.99, 95% CI [−31.48, −14.50], *I*^2^ = 93%, *P* < 0.00001) (Figure 9).

### 3.5. Sensitivity Analysis and Publication Bias

**Figure 10:**
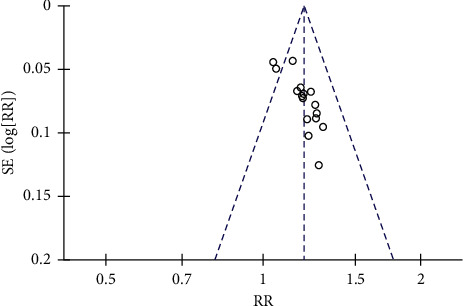
Funnel plot of total effective rate.

**Figure 11:**
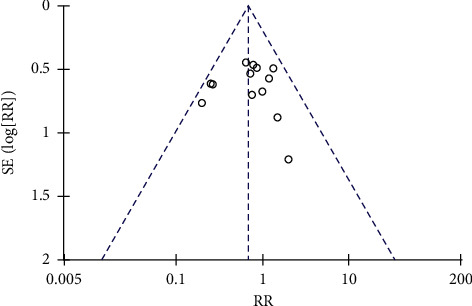
Funnel plot of adverse reaction rate.

A sensitivity analysis was performed based on the results of high heterogeneity. After removing the articles one by one, the heterogeneity results did not change significantly, indicating that the sensitivity of the whole analysis result is low. The funnel plot of the total effective rate is incompletely symmetrical, suggesting that there may be potential publication bias (Figure 10) and the funnel plot of the adverse reaction rate is roughly symmetrical (Figure 11).

### 3.6. Certainty of Evidence

The certainty of evidence was moderate, low, and very low. The result of the adverse reaction rate was moderate, and the total effective rate, recurrence rate, and IgE were low. The results of DLQI and IL-4 were very low. The main reasons for the downgrade were an unclear risk of bias and a small sample size. Details of the results are shown in Table 3.

## 4. Discussion

In the past, the systematic reviews of Chinese medicine in the treatment of CU focused more on acupuncture, while the clinical studies of Chinese medicine decoction or Chinese patent medicine were mostly small sample and single-center studies [50–52]. This may be related to the simple and easy operation of acupuncture. Acupuncture has a good effect on urticaria and is one of the treatment methods that many patients choose. Meanwhile, some patients worry about the pain and fear caused by acupuncture and choose Chinese herbal medicine for treatment. The RZZY capsule studied in this paper belongs to Chinese patent medicine. It not only has the effect of Chinese medicine decoction but also avoids the complexity of carrying Chinese medicine decoction. Systematic evaluation is helpful for further clinical application of Chinese patent medicine.

The routine medication method of the RZZY capsule is oral administration, 4 pills (0.5 g/pill) each time, three times a day for two weeks as a course of treatment. The above results suggested that the combined use of the RZZY capsule in the traditional treatment scheme is more beneficial for shortening the symptom duration of CU than simple conventional drugs, as well as obtaining better clinical efficacy, more effectively reducing the recurrence rate, and more significantly improving the quality of life of patients with a range (4–8 weeks) of treatment. Therefore, on the basis of antihistamines, combining RZZY capsule treatment could be a promising complementary therapy. Besides, relatively high safety is one of the potential advantages of CHM. In addition, IL-4 and IgE, as key substances causing allergic inflammation, can be effectively reduced by the RZZY capsule. At present, there are many patients with CU, which seriously reduces the life comfort of patients and aggravates the burden of individuals and society. Integrated Chinese and Western medicine treatment will be the most effective treatment for patients with CU.

Although this work has achieved some valuable results, there are several potential limitations in this review. Firstly, the studies included are all in Chinese, and the experimental studies were carried out in China. It is uncertain whether there will be the same research results in other countries or regions. Secondly, due to the relatively low-quality evaluation of research methods, there may be some implementation deviation. In addition, there is significant heterogeneity among the three outcome indicators, which may be related to the small sample size. The proof strength of the results may be affected by heterogeneity.

In view of the above-mentioned issues, the following suggestions are proposed: (a) the low quality of literature is mainly due to the defects of experimental design, such as random method, blind method, and sample size. Multicenter, large sample, and double-blind design should be adopted in future research, which is more meaningful to the systematic review. (b) Some articles did not have a clear follow-up time, which will be reported more clearly in future studies. (c) After the detailed study plan is formulated, it should be registered on the relevant website in advance and obtain research approval. Related websites include https://www.clinicaltrials.gov (https://www.clinicaltrials.gov), Chinese Clinical Trial Registry (ChiCTR, https://www.chictr.org/cn), etc.

## 5. Conclusion

Our study systematically evaluated the treatment of CU by the RZZY capsule, which will help more dermatologists to have a further understanding of traditional Chinese medicine and also provide more evidence-based medical evidence for the clinical use of the RZZY capsule. Despite the potential limitations of this review, meaningful conclusions were drawn through systematic reviews and meta-analyses based on randomized controlled trials. This will provide a reference for the clinical promotion and use of the Chinese patent medicine RZZY capsule and promote the treatment of CU. Future research still needs to be further confirmed by more large samples, multicenters, and high-quality articles.

## Figures and Tables

**Table 1:** Basic characteristics of included articles.

Author	Cases	Gender	Age	Treatment	Duration	Outcomes
	I/C	I/C	I/C	I/C	I/C	
Zhou [19]	55/53	(M31/F24)/	35.26 ± 1.37/	RZ + C/Ebastine	4 w	①②③④
		(M30/F23)	35.19 ± 1.31	10 mg po qd		

Zhang [20]	45/45	(M23/F22)/	40.25 ± 1.7/	RZ + C/Levocetirizine	28 d	①
		(M24/F21)	40.15 ± 1.25	5 mg po qd		

Chen et al. [21]	35/35	(M19/F16)/	39.08 ± 3.2/	RZ + C/Ebastine	4 w	①②④⑤⑥
		(M17/F18)	37.89 ± 4.35	10 mg po qd		

Ai [22]	24/24	(M14/F10)/	35.25 ± 1.39/	RZ + C/Levocetirizine	8 w	①②⑤
		(M13/F11)	35.12 ± 1.46	10 mg po qddsb		

Cheng [23]	50/50	(M28/F22)/	26.21 ± 2.11/	RZ + C/Epinastine	4 w	①②
		(M27/F23)	26.26 ± 2.24	10 mg po qd		

Li [24]	50/50	(M26/F24)/	48.56 ± 3.26/	RZ + C/Olopatadine	4 w	①②
		(M25/F25)	49.95 ± 3.86	5 mg po bid		

Li and Zhang [25]	45/45	(M27/F18)/	30.5 ± 4.2/	RZ + C/Levocetirizine	4 w	①
		(M25/F20)	25.6 ± 3.9	5 mg po qd		

Yang [26]	61/61	(M30/F31)/	31.8 ± 10.6/	RZ + C/Desloratadine	4 w	①②
		(M29/F32)	30.5 ± 9.8	5 mg po qd		

Liu and Yang [27]	50/50	(M28/F22)/	47.6/48.3	RZ + C/Levocetirizine	8 w	①②③
		(M31/F19)		5 mg po qd		

Bian et al. [28]	106/94	(M60/F46)/	34.93 ± 2.53/	RZ + C/Loratadine	4 w	①②③⑤⑥
		(M56/F38)	35.13 ± 2.15	10 mg po qd		

Lv [29]	59/59	(M29/F30)/	40.59 ± 11.91/	RZ + C/Ebastine	4 w	①②
		(M33/F26)	40.92 ± 12.02	20 mg po qd		

Li et al. [30]	35/35	(M18/F17)/	34.23 ± 9.86/	RZ + C/Fexofenadine	8 w	①②③
		(M19/F16)	33.12 ± 11.13	60 mg po bid		

Feng et al. [31]	60/60	(M32/F28)/	34.5/36.5	RZ + C/Desloratadine	4 w	①②③⑥
		(M24/F36)		Citrate disodium		
				8.8 mg po qd		

Xu et al. [32]	50/50	(M23/F27)/	33.5 ± 5.7/	RZ + C/Levocetirizine	4 w	①②⑥
		(M21/F29)	30.9 ± 6.7	5 mg po qd		

Xu et al. [33]	50/50	(M29/F21)/	33.9 ± 16.6/	RZ + C/Ebastine	4 w	①④
		(M27/F23)	33.1 ± 16.3	10 mg po bid/1^th^ W;		
				qd/2^th^ W; qod/3^th^ W;		
				3 times/4^th^ W		

Wu and Zhou [34]	68/66	(M35/F33)/	29.5/30.2	RZ + C/Epinastine	4 w	①②
		(M34/F32)		10 mg po qd		

Wang and Fang [35]	46/46	(M30/F16)/	35.8 ± 1.5/	RZ + C/Mizolastine	4 w	①②③
		(M28/F18)	35.7 ± 1.6	10 mg po qd		

I, intervention group; C, control group; M, male; F, female; RZ, Runzao Zhiyang capsule (2 g po tid); w, weeks; d, days; ① total effective rate; ② adverse reaction rate; ③ recurrence rate; ④ Dermatology Life of Quality Index; ⑤ IL-4 level; and ⑥ IgE level.

**Table 2:** Methodological quality evaluation of articles.

Author	Random sequence generation	Allocation concealment	Blind method	Incomplete outcome data	Selective reporting	Other bias
Zhou [19]	“Random” word	NM	Nonblind	N	N	N
Zhang [20]	Random number table	NM	NM	N	N	N
Chen et al. [21]	Random number table	NM	Nonblind	N	N	N
Ai [22]	Random touch ball	NM	Nonblind	N	N	N
Cheng [23]	Random number table	NM	Nonblind	N	N	N
Li [24]	Random number table	NM	Nonblind	N	N	N
Li and Zhang [25]	“Random” word	NM	NM	N	N	N
Yang [26]	“Random” word	NM	Nonblind	N	N	N
Liu and Yang [27]	“Random” word	NM	NM	N	N	N
Bian et al. [28]	“Random” word	NM	Nonblind	N	N	N
Lv [29]	“Random” word	NM	NM	N	N	N
Li et al. [30]	Random number table	NM	NM	N	N	N
Feng et al. [31]	Random number table	NM	Nonblind	N	N	N
Xu et al. [32]	According to the single and even number of visit tail number	NM	NM	2 cases were not followed up	N	N
Xu et al. [33]	According to the examining sequence	NM	Nonblind	N	N	N
Wu and Zhou [34]	According to the examining sequence	NM	NM	N	N	N
Wang and Fang [35]	Random number table	SAS system	Nonblind	N	N	N

N, no; NM, not mentioned.

**Table 3:** Summary of findings.

Outcomes	No of participants (studies) follow-up	Certainty of the evidence (GRADE)	Relative effect (95% CI)	Anticipated absolute effects	
				Risk with antihistamines	Risk difference with RZZY capsule + antihistamines
Total effective rate	1760	⊕⊕⃝⃝	RR 1.20	804 per 1,000	161 more per 1,000
	(17 RCTs)	Low^a,d^	(1.15 to 1.24)		(121 more to 193 more)

Adverse reaction rate	1482	⊕⊕⊕⃝	RR 0.68	127 per 1,000	41 fewer per 1,000
	(14 RCTs)	Moderate^a^	(0.50 to 0.92)		(63 fewer to 10 fewer)

Recurrence rate	503	⊕⊕⃝⃝	RR 0.29	246 per 1,000	174 fewer per 1,000
	(6 RCTs)	Low^a^	(0.18 to 0.46)		(201 fewer to 133 fewer)

Dermatology life of quality index	278	⊕⃝⃝⃝	—	—	MD 2.95 lower
	(3 RCTs)	Very low^a,b,c^			(4.32 lower to 1.57 lower)

Serum interleukin-4	318	⊕⃝⃝⃝	—	—	MD 13.83 lower
	(3 RCTs)	Very low^a,b,c^			(23.45 lower to 4.2 lower)

Immunoglobulin E	488	⊕⊕⃝⃝	—	—	MD 22.99 lower
	(4 RCTs)	Low^a,c^			(31.48 lower to 14.5 lower)

^*∗*^The risk in the intervention group (and its 95% confidence interval) is based on the assumed risk in the comparison group and the relative effect of the intervention (and its 95% CI).CI: confidence interval; MD: mean difference; RR: risk ratio. GRADE working group grades of evidence. High certainty: we are very confident that the true effect lies close to that of the estimate of the effect. Moderate certainty (⊕⊕⊕⃝): we are moderately confident in the effect estimate: the true effect is likely to be close to the estimate of the effect, but there is a possibility that it is substantially different. Low certainty (⊕⊕⃝⃝): our confidence in the effect estimate is limited: the true effect may be substantially different from the estimate of the effect. Very low certainty (⊕⃝⃝⃝): we have very little confidence in the effect estimate: the true effect is likely to be substantially different from the estimate of effect. Explanations: ^a^the blinding method was not used. ^b^number of patients included was less than 400. ^c^*I* square value was large. ^d^publication bias strongly suspected. RZZY capsule combined with conventional drugs compared to conventional drugs for people with chronic urticaria. Patient or population: patients with chronic urticaria. Intervention: RZZY capsule combined with antihistamines. Comparison: antihistamines.

## Data Availability

The data that support the findings of this study are available from the corresponding author upon reasonable request.

## Supplementary Materials

Supplementary Materials Additional file 1: search strategies of PubMed and CNKI.

## References

[1] Antia C., Baquerizo K., Korman A., Bernstein J. A., Alikhan A. (2018). Urticaria: a comprehensive review: epidemiology, diagnosis, and work-up. *Journal of the American Academy of Dermatology*.

[2] Kudryavtseva A. V., Neskorodova K. A., Staubach P. (2019). Urticaria in children and adolescents: an updated review of the pathogenesis and management. *Pediatric Allergy & Immunology*.

[3] Gonçalo M. A., Gimenéz-Arnau A., Al-Ahmad M. (2021). The global burden of chronic urticaria for the patient and society. *British Journal of Dermatology*.

[4] Fricke J., Ávila G., Keller T. (2020). Prevalence of chronic urticaria in children and adults across the globe: systematic review with meta-analysis. *Allergy*.

[5] Ghazanfar M. N., Kibsgaard L., Thomsen S. F., Vestergaard C. (2020). Risk of comorbidities in patients diagnosed with chronic urticaria: a nationwide registry-study. *World Allergy Organization Journal*.

[6] Memet B., Vurgun E., Barlas F., Metz M., Maurer M., Kocatürk E. (2021). In chronic spontaneous urticaria, comorbid depression linked to higher disease activity, and substance P levels. *Frontiers in Psychiatry*.

[7] Konstantinou G. N., Konstantinou G. N. (2019). Psychiatric comorbidity in chronic urticaria patients: a systematic review and meta-analysis. *Clinical and Translational Allergy*.

[8] Staubach P., Eckhardt-Henn A., Dechene M. (2006). Quality of life in patients with chronic urticaria is differentially impaired and determined by psychiatric comorbidity. *British Journal of Dermatology*.

[9] Dias G. A. C., Pires G. V., Valle S. O. R. d. (2016). Impact of chronic urticaria on the quality of life of patients followed up at a university hospital. *Anais Brasileiros de Dermatologia*.

[10] Zuberbier T., Aberer W., Asero R. (2018). The EAACI/GA2LEN/EDF/WAO guideline for the definition, classification, diagnosis and management of urticaria. *Allergy*.

[11] Passanisi S., Arasi S., Caminiti L., Crisafulli G., Salzano G., Pajno G. B. (2020). Omalizumab in children and adolescents with chronic spontaneous urticaria: case series and review of the literature. *Dermatologic Therapy*.

[12] Khan D. A. (2013). Alternative agents in refractory chronic urticaria: evidence and considerations on their selection and use. *Journal of Allergy and Clinical Immunology: In Practice*.

[13] Kitsioulis N. A., Xepapadaki P., Roussaki-Schulze A. V., Papadopoulos N., Zafiriou E. (2017). Effectiveness of autologous whole-blood injections in patients with refractory chronic spontaneous urticaria. *International Archives of Allergy and Immunology*.

[14] Yang S. H., Lin Y. H., Lin J. R. (2018). The efficacy and safety of a fixed combination of Chinese herbal medicine in chronic urticaria: a randomized, double-blind, placebo-controlled pilot study. *Frontiers in Pharmacology*.

[15] Liao Y. J., Liao K. F., Chen F. P. (2020). The efficacy of patients with moderate to severe atopic dermatitis treated with Chinese herbal medicine. *Integrative Medicine Research*.

[16] Coyle M. E., Yu J. J., Zhang A. L., Jones L., Xue C. C., Lu C. (2020). Patient experiences of using Chinese herbal medicine for psoriasis vulgaris and chronic urticaria: a qualitative study. *Journal of Dermatological Treatment*.

[17] Wang Y. m., Du L., Zhu Y. j. (2016). Evidence-based therapies of Chinese medicine for chronic urticaria: where do we stand and where are we going?. *Chinese Journal of Integrative Medicine*.

[18] Chen H. Y., Lin Y. H., Huang J. W., Chen Y. C. (2015). Chinese herbal medicine network and core treatments for allergic skin diseases: implications from a nationwide database. *Journal of Ethnopharmacology*.

[19] Zhou P. F. (2020). Discussion on the effectiveness of ebastine combined with Runzao Zhiyang capsule in the treatment of chronic urticaria. *Medical Diet and Health*.

[20] Zhang J. (2020). Therapeutic effect of Runzao Zhiyang capsule combinedwith levocetirizine on chronic urticaria. *Smart Healthcare*.

[21] Chen Z. M., Wu S. R., Ge W. W. (2020). Effect of Runzao Zhiyang capsule and ebastine on chronic urticaria and levels of immune factors. *Chinese Archives of Traditional Chinese Medicine*.

[22] Ai H. P. (2020). Therapeutic effect of Runzao Zhiyang capsule combined with levocetirizine on chronic urticaria. *Drug Evaluation*.

[23] Cheng Y. L. (2019). The effect of epistine combined with Runzao Zhiyang capsule on chronic urticaria. *China Continuing Medical Education*.

[24] Li T. H. (2019). Clinical efficacy of Runzao Zhiyang Capsule combined with loratadine hydrochloride in the treatment of chronic urticaria. *The Medical Forum*.

[25] Li J. P., Zhang Z. (2019). Efficacy analysis of Runzao Zhiyang Capsule combined with levocetirizine in the treatment of chronic urticaria. *Electronic Journal of Clinical Medical Literature*.

[26] Yang J. (2019). Observation on the efficacy of Desloratadine tablets combined with Runzao Zhiyang Capsule in the treatment of chronic spontaneous urticaria. *Contemporary Medical Symposium*.

[27] Liu H. D., Yang M. (2019). Observation on the efficacy of Runzao Zhiyang Capsule combined with Levocetirizine in the treatment of chronic urticaria. *Health Guide*.

[28] Bian K. P., Xu B. L., Yang A. Q., Liang L. L. (2018). Efficacy of Runzao Zhiyang Capsule in the treatment of chronic urticaria and its effect on peripheral blood CD4 + /CD8 + level. *Shaanxi Zhongyi*.

[29] Lv J. (2018). Runzao Zhiyang Capsule combined with double dose Ebastine in the treatment of refractory chronic idiopathic urticaria. *Journal of Hubei University for Nationalities·Medical Edition*.

[30] Li Y. X., Han T. M., Lu J. (2017). Clinical observation of Fexofenadine combined with Runzao Zhiyang Capsule in the treatment of chronic urticaria of blood deficiency and wind dryness. *Chinese Journal of Dermatology and Venereology of Integrated Traditional Chinese and Western Medicine*.

[31] Feng H. X., Kang C. Y., Guo G. (2016). Effect of Runzaozhiyang Capsule combined with desloratadine citrate disodium Tablets in treatment of chronic urticaria and its influence on total serum IgE levels. *Journal of Disease Monitor and Control*.

[32] Xu H., Ma H., Li Y. M. (2016). Effect of Runzao Zhiyang Capsule combined with Levocetirizine on patients with chronic spontaneous urticaria. *Chinese Journal of Dermatology and Venereology*.

[33] Xu H., Xu M. X., Hu Y. J. (2014). Observation on the efficacy of Ebastine tablet decreasing therapy combined with Runzao Zhiyang Capsule in the treatment of chronic idiopathic urticaria. *Medical Information*.

[34] Wu Z. Q., Zhou P. H. (2013). Observation on the efficacy of Runzao Zhiyang Capsule combined with Epistatin in the treatment of chronic urticaria. *Diagnosis and Therapy Journal of Dermato-Venereology*.

[35] Wang F., Fang L. L. (2013). Clinical study of Mizolastine combined with traditional Chinese medicine in the treatment of chronic idiopathic urticaria. *Chinese Journal of Primary Medicine and Pharmacy*.

[36] Mei X., Yu L. Q., Chen X. Y., Zhou C. Y. (2016). Advances in studies on the chemical components and pharmacological activities of Radix Polygoni multiflora. *Drug Evaluation Research*.

[37] Xue X. Y., Quan Y. Y., Gong L. H., Gong X. H., Li Y. X. (2020). A review of the processed *Polygonum multiflorum* (Thunb.) for hepatoprotection: clinical use, pharmacology and toxicology. *Journal of Ethnopharmacology*.

[38] Lin Y., Xiao R., Li C. (2018). Research progress on chemical constituents,pharmacology and hepatoxicity of raw,processed and fermented polygonum multiflorum thunb. *Traditional Chinese Drug Research & Clinical Pharmacology*.

[39] Luo C. Y., Qin S. Z., Liang P. L., Ling W. P., Zhao C. G. (2020). Advances in modern research on pharmacological effects of radix rehmanniae. *AIP Conference Proceedings*.

[40] Xu L., Zhang W., Zeng L., Jin J. O. (2017). Rehmannia glutinosa polysaccharide induced an anti-cancer effect by activating natural killer cells. *International Journal of Biological Macromolecules*.

[41] Xu L., Kwak M., Zhang W., Zeng L., Lee P. C., Jin J. O. (2017). Rehmannia glutinosa polysaccharide induces toll-like receptor 4-dependent spleen dendritic cell maturation and anti-cancer immunity. *Oncolmmunology*.

[42] Liu L., Tang L., Xu D. S., Xia H. L., Xie Q. M. (2007). Effect of Shengdi injection on lipopolysaccharide induced pulmonary inflammation in rats. *China Journal of Chinese Materia Medica*.

[43] Han X., Song C., Feng X. (2020). Isolation and hypoglycemic effects of water extracts from mulberry leaves in Northeast China. *Food & Function*.

[44] Lai L. L., Peng X. F., Leng E. N., Mo Z. T., Li W. N. (2016). Advances in study on pharmacological effects of Mulberry leaves. *Anhui Medical and Pharmaceutical Journal*.

[45] Su Z. Q., Zhao Z. Y., Xie S. N. (2009). Effects of analgesia, anti-inflammatory and immunosuppression of extracts of acetic ether extract of Chinese medicine honghuoma. *Chinese Pharmacological Bulletin*.

[46] Lu J. L., Li W. J., Hou W. R. (2012). Study on the effect of total coumarins of red live hemp on colitis induced by dextran sodium sulfate in mice. *China Journal of Chinese Materia Medica*.

[47] Page M. J., McKenzie J. E., Bossuyt P. M. (2021). The PRISMA 2020 statement: an updated guideline for reporting systematic reviews. *British Medical Journal*.

[48] Urticaria Research Center Society of Dermatology and Venereology and Chinese Medical Association (2019). Chinese guidelines for the diagnosis and treatment of urticaria (2018 Edition). *Chinese Journal of Dermatology*.

[49] Higgins J. P., Green S. (2011). *Cochrane Handbook for Systematic Reviews of Interventions*.

[50] Yao Q., Li S., Liu X., Qin Z., Liu Z. (2016). The effectiveness and safety of acupuncture for patients with chronic urticaria: a systematic review. *BioMed Research International*.

[51] Yao Q., Ye Y., Liu X., Qin Z., Liu Z. (2015). Acupuncture for patients with chronic urticaria: a systematic review protocol. *BMJ Open*.

[52] Hwang J., Lio P. A. (2021). Acupuncture in Dermatology: an update to a systematic review. *Journal of Alternative & Complementary Medicine*.

