# Supplementary material for: A Meta-Analysis of Randomized Clinical Trials of Runzao Zhiyang Capsule in Chronic Urticaria
Source: Evid Based Complement Alternat Med. 2022 Sep 17;2022:1904598. doi: 10.1155/2022/1904598 (PMC9509263; doi:10.1155/2022/1904598)
Supplement: Supplementary Materials — Additional file 1: search strategies of PubMed and CNKI. [file 1904598.f1.docx]

**Supplementary Materials**

**A Meta-Analysis of Randomized Clinical Trials of Runzao Zhiyang Capsule in Chronic Urticaria**

Sheng-Zhen Ye ^a,b^, Xue-Er Zhang ^a,b^,Gui-Hua Ling ^a,b^, Xian-Jun Xiao ^a^,Dan Huang ^a,b^, Ming-Ling Chen ^b,*^

^a^ Chengdu University of Traditional Chinese Medicine, Chengdu, China

^b^ Affiliated Hospital of Chengdu University of Traditional Chinese Medicine, Chengdu, China

**Search strategies**

**1.The search strategy of PubMed**

Search: **((("Chronic Urticaria"[Mesh]) OR ((((((((((((((((((((((((((Chronic Urticarias[Title/Abstract]) OR (Urticaria, Chronic[Title/Abstract])) OR (Chronic Inducible Urticaria[Title/Abstract])) OR (Chronic Inducible Urticarias[Title/Abstract])) OR (Inducible Urticaria, Chronic[Title/Abstract])) OR (Urticaria, Chronic Inducible[Title/Abstract])) OR (CIndU[Title/Abstract])) OR (Chronic Spontaneous Urticaria[Title/Abstract])) OR (Chronic Spontaneous Urticarias[Title/Abstract])) OR (Spontaneous Urticaria, Chronic[Title/Abstract])) OR (Urticaria, Chronic Spontaneous[Title/Abstract])) OR (Idiopathic Chronic Urticaria[Title/Abstract])) OR (Chronic Urticaria, Idiopathic[Title/Abstract])) OR (Idiopathic Chronic Urticarias[Title/Abstract])) OR (Urticaria, Idiopathic Chronic[Title/Abstract])) OR (Chronic Idiopathic Urticaria[Title/Abstract])) OR (Chronic Idiopathic Urticarias[Title/Abstract])) OR (Idiopathic Urticaria, Chronic[Title/Abstract])) OR (Urticaria, Chronic Idiopathic[Title/Abstract])) OR (Autoimmune Urticaria[Title/Abstract])) OR (Autoimmune Urticarias[Title/Abstract])) OR (Urticaria, Autoimmune[Title/Abstract])) OR (Chronic Autoimmune Urticaria[Title/Abstract])) OR (Autoimmune Urticaria, Chronic[Title/Abstract])) OR (Chronic Autoimmune Urticarias[Title/Abstract])) OR (Urticaria, Chronic Autoimmune[Title/Abstract]))) AND (randomized controlled trial[Publication Type] OR (randomized[Title/Abstract] AND controlled[Title/Abstract] AND trial[Title/Abstract]))) AND (Runzao Zhiyang Capsule[Title/Abstract])**

**2.The search strategy of** **China National Knowledge Infrastructure (CNKI)**

**（主题：慢性荨麻疹（精确））OR （主题：荨麻疹（精确））AND （主题：润燥止痒胶囊（精确））AND （（摘要：随机对照（精确））OR （摘要：随机（精确））OR （摘要：RCT（精确））**

**(subject: chronic urticaria (accurate)) or (subject: urticaria (accurate)) and (subject: Runzao Zhiyang capsule (accurate)) and (Abstract: randomized control (accurate)) or (Abstract: randomized (accurate)) or (Abstract: RCT (accurate))**
